# Steaming duration-dependent effects on the *in vivo* distribution, anti-fatigue activity, and gut microbiota modulation of *Polygonatum cyrtonema* Hua polysaccharides

**DOI:** 10.3389/fphar.2025.1721319

**Published:** 2026-01-07

**Authors:** Shengrong Lin, Yanling Wang, Weijing Wu, Jiaqi Huang, Xinqi Cai

**Affiliations:** 1 Orthopedic Department, First Affiliated Hospital of Xiamen University, Xiamen, Fujian, China; 2 School of Public Health and Medical Technology, Xiamen Medical College, Xiamen, China; 3 Engineering Research Center of Natural Cosmeceuticals College of Fujian Province, Xiamen Medical College, Xiamen, China

**Keywords:** anti-fatigue activities, gut microbiota, In vivo distribution, network pharmacology, Polygonatum cyrtonema Hua, polysaccharides, steaming processing

## Abstract

**Background:**

*Polygonatum cyrtonema* Hua is a traditional medicinal plant widely used for its anti-fatigue properties, with its polysaccharides (PCPs) believed to play a key role in these effects. Steaming, a necessary step before consumption, can alter the structure of PCPs. However, the impact of steaming-induced structural changes on their anti-fatigue activity remains unexplored. This study aims to investigate how different steaming durations affect the distribution, anti-fatigue activity, and modulation of gut microbiota by PCPs.

**Methods:**

*P. cyrtonema* rhizomes were steamed for 0, 4, 8, and 12 h, and the polysaccharides were extracted. *In vivo* distribution was evaluated by conjugating PCPs with fluorescein isothiocyanate (FITC) for optical imaging. Anti-fatigue effects were assessed via exhaustive swimming tests, and serum biochemical markers were analyzed. Short-chain fatty acids (SCFAs) in feces were quantified by gas chromatography, while gut microbiota composition was analyzed using 16S rRNA sequencing. Network pharmacology analysis was performed to identify the biological targets of PCPs and their role in fatigue.

**Results:**

The results showed that all four PCPs exhibited poor systemic absorption but prolonged colonic retention, suggesting that interactions with the gut microbiota are central to their biological effects. While all PCPs improved exercise endurance, the 12-h steaming treatment notably decreased biochemical markers such as lactate and lactate dehydrogenase levels. Microbiota analysis revealed increased gut microbial α-diversity and short-chain fatty acids (SCFAs), with the 4-h and 8-h steaming groups showing the highest SCFA levels. β-diversity analysis indicated distinct microbial shifts, particularly between raw and steamed samples. Network pharmacology analysis highlighted differences in the mechanisms between raw and steamed PCPs, identifying key biological targets related to the anti-fatigue effects. The analysis suggested that the duration of steaming affects the biological activity of PCPs by modulating key metabolic pathways and targets involved in fatigue.

**Conclusion:**

These findings suggest that steaming-induced structural changes affect function and that the duration of steaming plays a crucial role in preserving the anti-fatigue effects of *P. cyrtonema* rhizomes. The results provide valuable insights for optimizing the steaming process to enhance the anti-fatigue potential of *P. cyrtonema*.

## Graphical Abstract

**Figure:**
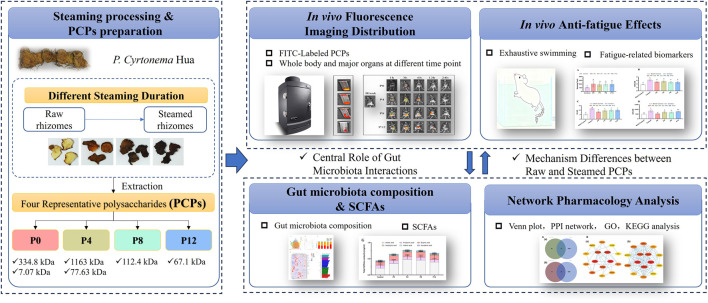

## 1 Introduction

Fatigue has become a prevalent issue in modern societies, causing a range of physiological impairments. *Polygonatum Cyrtonema* Hua has been used in traditional Chinese medicine and functional foods for centuries (Li X.-L. et al., 2021; Hu et al., 2023; Zhao et al., 2018). *P*. *cyrtonema* is regarded as a “Top-grade” medicinal herb in Shennong Bencao Jing, historically prescribed for invigorate Qi, nourish Yin, moisten lung, and tonify spleen and kidney (Liu S. et al., 2022; Wang J. et al., 2022). The rhizomes of *P. Cyrtonema* are the part used for consumption. Contemporary pharmacological investigations have demonstrated that *Polygonatum* exhibit multifunctional bioactivities. *P*. *Cyrtonema* has demonstrated high efficacy in clinical practice for treating fatigue (Zhao et al., 2018). The anti-fatigue effects of *P. cyrtonema* are primarily attributed to the neutral polysaccharide fraction (PCPs) in its rhizomes, which contributes to the regulation of osteocalcin signaling (Shen et al., 2021).

However, raw *Polygonatum* rhizomes, being inedible, can cause throat and tongue irritation, necessitating steaming before consumption. The traditional ‘nine steaming and nine drying’ process, which typically involves 6–12 h of steaming per cycle, is highly energy-intensive and raises substantial energy-consumption concerns (Chinese Pharmacopoeia Commission, 2020; Sun et al., 2020; Jin et al., 2018; Fan et al., 2020; Li Q. et al., 2021; Su et al., 2023). Our previous studies have shown steaming reduces the polysaccharide content and induces composition and structural modifications in PCPs, which, in turn, affect their biological activities (Wu et al., 2022; Wu et al., 2024). Steaming for 4 h shifts the molecular weight distribution of PCPs from two peaks in P0 (334.8 kDa and 7.07 kDa) to much larger peaks in P4 (1163kDa and 77.63 kDa). As steaming progresses, the molecular weight distribution further evolves in P8 and P12, where the profiles show progressively degraded single peaks at 112.4 kDa and 67.1 kDa, respectively (Li Q. et al., 2021). These changes highlight the gradual restructuring and breakdown restructuring of molecular weight distribution as steaming time increases. Carbohydrate content decreased markedly with steaming, while uronic acid increased (Wu et al., 2022). Steaming also altered monosaccharide composition, with P4 enriched in xylose, P0 containing more glucose derived from fructose, and P8/P12 showing elevated galactose levels (Wu et al., 2022). Additionally, steaming induces structural changes in PCPs that affect their immunological activities. Steaming for 4 h enhanced immunological activities, while steaming for 8 and 12 h causes excessive degradation, reducing immunological effectiveness and impairing the human gut microbiota fermentation (Wu et al., 2022; Wu et al., 2024). In addition, an *in vivo* immune activity study further supported that PCPs steamed for 6 cycles showed higher immune activity than those steamed for 9 cycles (Su et al., 2023). These findings highlight that the steaming duration significantly influences the structural and biological properties of PCPs, yet the impact of these changes on the anti-fatigue effects of PCPs remains unclear.

Furthermore, the molecular weight of polysaccharides plays a key role in their distribution patterns within the gut, affecting gut microbiota fermentation. Lower molecular weight polysaccharides can be absorbed and target specific organs (Zhang B. et al., 2021; Wang et al., 2017), while higher molecular weight polysaccharides are indigestible and non-absorbing, ultimately modulating gut microbiota (Li et al., 2019). Different steaming duration resulted in varying molecular weight fractions of PCPs (Wu et al., 2022), but how these variations influence gut microbiota modulation and anti-fatigue effects remains poorly understood. Although targeting gut microbiota has been suggested as a strategy for developing anti-fatigue foods (Li Y. et al., 2021; Zhou et al., 2023), the mechanisms behind this modulation, particularly in relation to anti-fatigue effects, have not been thoroughly investigated.

Building on our previous findings, four PCPs from rhizomes steamed for different durations (0, 4, 8, and 12 h), each showing significant variations in molecular weight and monosaccharide composition, were selected for this study. This study aimed to compare their *in vivo* distribution and anti-fatigue effects, investigate the relationship between gut microbiota and fatigue-related biomarkers, Additionally, network pharmacology was used to explore the mechanisms behind steaming-induced differences in anti-fatigue activity induced by steaming.

## 2 Materials and methods

### 2.1 Material and reagents

The rhizomes of *P. Cyrtonema* Hua (wild-type) were collected in October from Shaowu, Fujian, China (Supplementary Figure S1). The botanical identity was authenticated by associate professor Qing Wang, Xiamen Medical College, Fujian, China. A voucher specimen (No. XMMC-2022–05) has been deposited in the Xiamen Medical College. The raw material complied with the quality requirements outlined in the Chinese Pharmacopoeia (2020 edition). Short-chain fatty acids (≥99.5%) were purchased from Aladin Company (Shanghai, China). All other chemicals used were of analytical grade. Commercial kits of lactate dehydrogenase (LDH), lactic acid (BLA), blood urea nitrogen (BUN) were purchased from Nanjing Jiancheng Bioengineering Institute (Nanjing, Jiangsu Province, China)

### 2.2 Processing of *P. Cyrtonema* and extraction of PCPs

Polysaccharides were extracted from dried *P. cyrtonema* Hua rhizomes, which had been steamed at 100 °C under atmospheric pressure, with no additional excipients, for 4, 8, and 12 h, respectively (Wu et al., 2022). Steaming was performed in an ACA steaming chamber with a continuous water vapor supply under atmospheric pressure. The raw rhizome without steaming served as the control. The powdered samples were defatted with 95% ethanol, followed by extraction with distilled water at a drug-extract ratio of 1:10 (w/v) at 80 °C for 1 h. After centrifugation, the supernatant was collected, and PCPs were precipitated by adding 4 volumes of anhydrous ethanol to the supernatant. The precipitate was collected by centrifugation at 5,000 r/m for 2 min, then re-dissolved in water. Insoluble particles were removed by centrifugation (10,000 r/m, 30 min), and the resulting supernatant was lyophilized. PCPs were obtained from raw rhizomes (P0) and rhizomes steamed for different durations (P4, P8, and P12). Different PCPs were store at 4 °C and used in subsequent analyses. A complete compositional profile of the PCPs was provided in previous studies, including the monosaccharide composition, molecular weight distribution, FT-IR characteristics (Wu et al., 2022), and the contents of carbohydrates, uronic acids, polyphenols, and proteins (Wu et al., 2024). Briefly, the carbohydrate content was 84.87% for P0, 59.82% for P4, 48.25% for P8, and 49.58% for P12. The uronic acid content was 13.99% for P0, 19.36% for P4, 19.57% for P8, and 19.61% for P12. The protein content was 1.07% for P0, 1.12% for P4, 2.64% for P8, and 5.43% for P12. The polyphenol content was 0.05% for P0, 0.47% for P4, 2.75% for P8, and 2.23% for P12. The molecular weight distribution showed peaks at 334.8 kDa and 7.07 kDa for P0, at 1,163 kDa and 77.63 kDa for P4, at 112.4 kDa for P8, and at 67.1 kDa for P12. The monosaccharide composition of P0 was mainly glucose and xylose, while P4 was primarily composed of xylose. P8 and P12 contained mainly galactose, and after steaming, P4 to P12 all contained galacturonic acid.

### 2.3 *In vivo* distribution of FITC-labeled PCPs

PCPs were conjugated with tyramine (TYR) and labeled with FITC following a procedure from previous studies (Wu et al., 2024). PCPs (400 mg) were dissolved in 15 mL phosphate buffer (pH 8.0), then reacted with 400 mg tyramine and 150 mg sodium cyanoborohydride at 37 °C for 96 h. After centrifugation and freeze-drying, the PCP-TYR was dissolved in Na_2_CO_3_ (pH 8.5) and labeled with 25 mg FITC in DMSO, stirred in the dark for 24 h, precipitated with ethanol, centrifuged, and washed. The final FITC-Labeled PCPs was freeze-dried, and the degree of FITC substitution was determined by fluorescence intensity at 490 nm excitation and 515 nm emission. Fluorescence characteristics, including intensity, stability, and substitution degree, were evaluated as previous described. Cellular experiments were also conducted to validate the labeling efficiency.

Male C57BL/6 mice (7–8 weeks old, 25 ± 1.5 g) were purchased from Wu’s Laboratory Animal Trading Co., Ltd. (Fuzhou, China). All animal experiments were conducted in accordance with the ethical standards and guidelines outlined in the Guide for the Care and Use of Laboratory Animals (NIH, United States) and were approved by the Medical Ethics Committee of Xiamen Medical College (Approval No. 20220901014). The animals were randomly divided into a blank control group and several PCP-Tyr-FITC groups, with five mice per group. The control group received an oral gavage of distilled water (1% of body weight). Each PCP-Tyr-FITC group received a single oral gavage of 5 mg of the corresponding FITC-labeled polysaccharide solution (calculated based on PCP), at a volume equivalent to 1% of body weight, resulting in a final dose of 200 mg/kg. At 1, 3, 6, 12, and 24 h after dosing, at each time point, 1 mouse from each group was anesthetized with isoflurane and subjected to *in vivo* optical imaging using a small-animal imaging system (Lumina XRMS, PerkinElmer, United States) to monitor the biodistribution of fluorescent polysaccharides. Whole-body fluorescence imaging was first performed, followed by organ-specific imaging of the heart, liver, kidney, spleen, lung, stomach, small intestine, and cecum/colon. Imaging parameters were set at an excitation wavelength of 490 nm and an emission wavelength of 515 nm. Fluorescence signals were normalized against the blank control group to eliminate background interference.

### 2.4 Animals and experimental groups

Six-week-old male specific pathogen-free ICR mice (n = 80) were obtained from Zhejiang Medical College (Zhejiang, China). After a 1-week acclimatization, the mice were randomly divided into 5 groups (16 mice/group) and named as follows: Control, P0, P4, P8, and P12. The control group was given the AIN 93 diet. The treatment group was given the AIN-93 diets (Reeves et al., 1993) supplemented with different PCPs (0.1% w/w). Additionally, a separate Blank Control group (n = 8) was housed under the same conditions as the Control group, but without undergoing swimming training, and was used for biochemical marker analysis. The treatment lasted for 8 weeks. Food intake, water intake, and weight were measured every week. The mice were given free access to water and diets with a 12-h light/dark cycle. The use of animals and experimental methods were in compliance with the National Institutes of Health Guidelines for the Care and Use of Laboratory Animals, approved by Xiamen Medical College (Approval No. 20220901014).

### 2.5 Exhaustive swimming test

Exhaustive swimming test was conducted according to previous study (Shen et al., 2021), with minor modifications. Prior to weigh-loaded swimming test, the mice underwent a training session once a week. Before the test, the mice were fasted for 2h, then 8 mice of each group were place in the tank (45 cm × 35 cm × 35 cm), water temperature of 25 °C with a lead block (weighing 5% of the body weight) attached to its tail and forced to swim until exhaustion. The exhaustion criterion was failure to resurface within 8 s.

### 2.6 Evaluation of biochemical markers for anti-fatigue

Following the procedure described in a previous study (Jiang et al., 2023; Wang et al., 2020) with minor modifications, the remaining 8 mice from each group were allowed to swim without the added weight for 30 min, followed by 10 min of rest. Additionally, mice in the blank control group did not undergo swimming and were anesthetized directly to serve as the baseline resting control for biochemical markers. Afterward, the mice were anesthetized by gradual carbon dioxide. Serum was separated from the collected blood by centrifugation at 3,000 r/min, 4 °C for 10 min. LDH, BLA, and BUN levels in the serum were measured using commercial kits, according to the manufacturer’s instructions.

### 2.7 Determination of short-chain fatty acids (SCFAs)

The measurement of SCFAs in feces was performed according to our previous study (Wu et al., 2021). The mice were placed in individual cages for 1 hour in the morning. Then, the feces of mice were collected (8 mice/group), weighed, and mixed with acidified water (pH 2, prepared with H_2_SO_4_) at a ratio of 1:5 (w/v) at 4 °C for 30 min, with vertexing every 10 min. The mixture was centrifuged at 10,000 r/min for 20 min at 4 °C. The supernatant was filtered through a 0.22 μm filter. SCFAs were analyzed by gas chromatography (Agilent 7890B GC system equipped with a flame ionization detector). The SCFAs were separated on an HP INNO-WAX (ID 25mm, length 30 m, 0.25 μm film thickness, JW scientific, United States) using the following program. The initial temperature was 100 °C. The injection was 1 μL. It was raised to 125 °C by 3 °C/min, increased to 200 °C by 20 °C/min, and held at 230 °C for 2 min. The injection port was operated at 250 °C. The detector temperature was 280 °C. The carrier gas was nitrogen at 25 mL/min. Sample quantification was performed using standard SCFAs, and the standard curve equations are provided in Supplementary Table S1. The fecal SCFA content was calculated according to the standard formulas.

### 2.8 16S rRNA sequencing

#### 2.8.1 DNA extraction and amplification

Colon content samples were frozen by liquid nitrogen after collection and stored at −80 °C (n = 6/group). Bacterial DNA was isolated from the colon contents using a MagPure Soil DNA LQ Kit (Magen, Guangdong, China). DNA concentration and integrity were measured by a NanoDrop 2000 spectrophotometer (Thermo Fisher Scientific, Waltham, MA, United States) and agarose gel electrophoresis, respectively. PCR amplification of the V3-V4 hypervariable regions of the bacterial 16S rRNA gene was carried out in a 25 μL reaction using universal primer pairs (343F: 5′-TACGGRAGGCAGCAG-3′; 798R: 5′-AGG​GTA​TCT​AAT​CCT-3′). The reverse primer contained a sample barcode and both primers were connected with an Illumina sequencing adapter.

#### 2.8.2 Library construction and sequencing

The Amplicon quality was visualized using gel electrophoresis. The PCR products were purified with Agencourt AMPure XP beads (Beckman Coulter Co., United States) and quantified using Qubit dsDNA assay kit. The concentrations were then adjusted for sequencing. Sequencing was performed on an Illumina NovaSeq6000 with two paired-end read cycles of 250 bases each. (Illumina Inc., San Diego, CA; OE Biotech Company; Shanghai, China).

#### 2.8.3 Bioinformatic analysis

The library sequencing and data processing were conducted by OE biotech Co., Ltd. (Shanghai, China). Paired-end reads were then preprocessed using Trimmomatic software to detect and cut off ambiguous bases (N) (Bolger et al., 2014). It also cut off low quality sequences with average quality score below 20 using sliding window trimming approach. After trimming, paired-end reads were assembled using FLASH software (Reyon et al., 2012). Parameters of assembly were: 10bp of minimal overlapping, 200bp of maximum overlapping and 20% of maximum mismatch rate. Sequences were performed further denoising as follows: reads with ambiguous, homologous sequences or below 200bp were abandoned. Reads with 75% of bases above Q20 were retained. Then, reads with chimera were detected and removed. These two steps were achieved using QIIME software (version 1.8.0) (Caporaso et al., 2010). Clean reads were subjected to primer sequences removal and clustering to generate operational taxonomic units (OTUs) using Vsearch software with 97% similarity cutoff (Rognes et al., 2016). The representative read of each OTU was selected using QIIME package. All representative reads were annotated and blasted against Silva database Version 138 (16s/18s rDNA) using RDP classifier (confidence threshold was 70%) (Rognes et al., 2016). All representative reads were annotated and blasted against Unite database (ITSs rDNA) using BLAST (Bolger et al., 2014; Altschul et al., 1990).

### 2.9 Network pharmacology analysis

The SMILES for monosaccharide constituents within both raw and steamed PCPs were retrieved from the PubChem database (https://pubchem.ncbi.nlm.nih.gov), and then the SMILES were used to predict potential biological targets through three platforms: the Similarity Ensemble Approach (SEA, https://sea.bkslab.org), PharmMapper (https://www.lilab-ecust.cn/pharmmapper/submitfile.html), and the SwissTargetPrediction database (http://swisstargetprediction.ch). Targets were standardized using the UniProt database (https://www.uniprot.org), followed by removal of duplicate entries, resulting in the identification of putative targets for PCPs. A search for “fatigue” was conducted in the GeneCards database (https://www.genecards.org). These fatigue-related targets were also standardized using UniProt and any duplicates were removed. The common targets between PCPs and fatigue were then identified and visualized using a Venn diagram generated with a bioinformatics online platform (http://www.bioinformatics.com.cn).

The common targets were then imported into the STRING database (https://cn.string-db.org) for Protein-Protein Interaction (PPI) network analysis, with the species parameter restricted to “*Homo sapiens*”. The “tsv” file that was produced was imported into Cytoscape 3.10.3 for additional analysis after the study was carried out with the maximum confidence score set at 0.400, and hub genes were identified using the cytoHubba plugin based on topological analysis algorithms. Functional enrichment analysis of the shared targets was performed using the DAVID database (https://david.ncifcrf.gov). Finally, the “Functional Annotation” tool was employed with the species set as *H. sapiens* and a significance threshold of *P* < 0.05. The results for Gene Ontology (GO) terms and Kyoto Encyclopedia of Genes and Genomes (KEGG) pathways were graphically represented as a bar chart and a bubble plot, respectively, using the aforementioned bioinformatics platform (http://www.bioinformatics.com.cn).

### 2.10 Statistical analysis

The data are expressed as the means with standard deviations (SD). Statistical differences among groups were evaluated by one-factor analysis of variance (ANOVA) and Duncan’s test for *post hoc* analysis. Data were statistically significant at *P* < 0.05.

## 3 Results

### 3.1 Oral absorption mechanism of different PCPs based on fluorescence labeling by FITC

**FIGURE 1:**
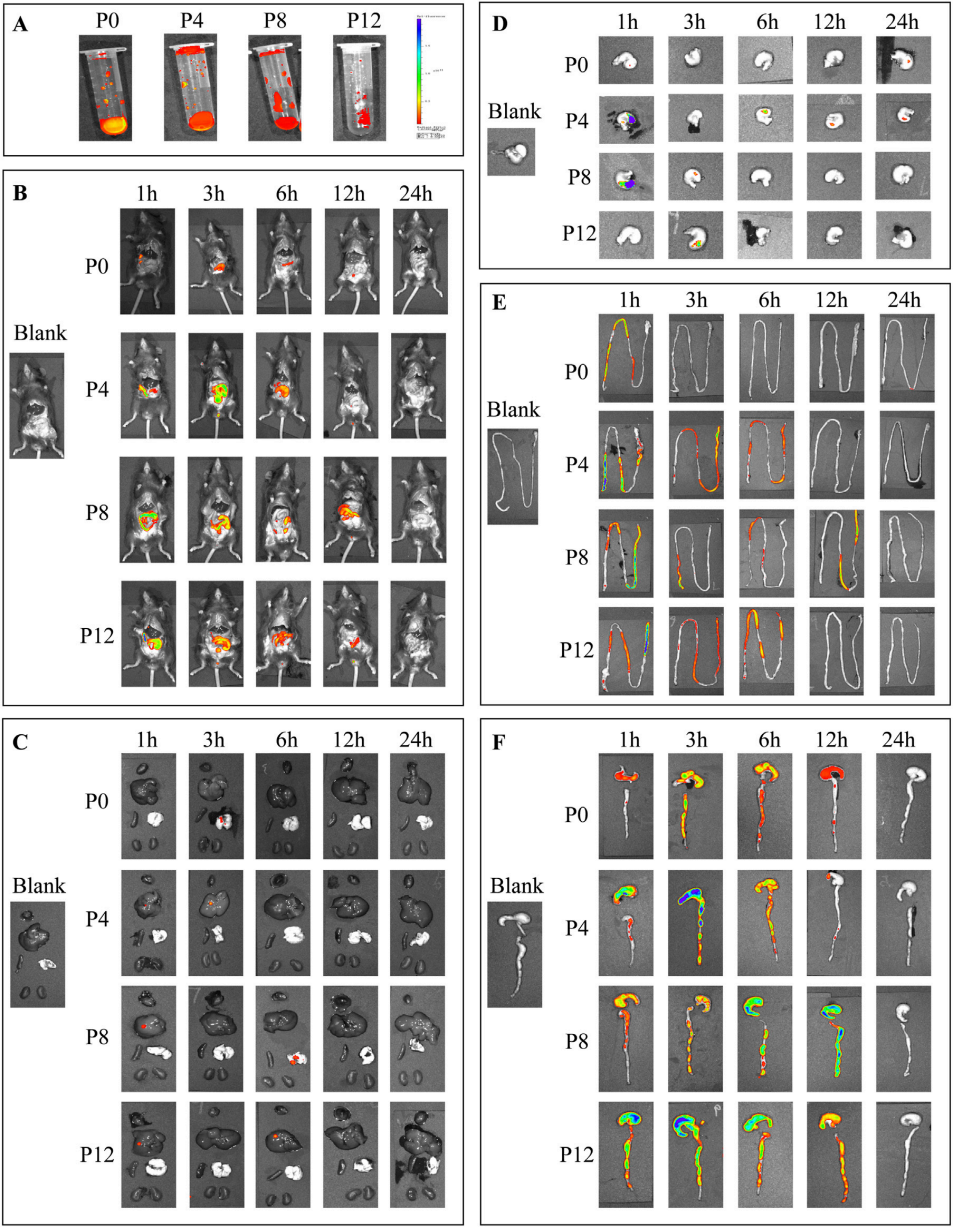
Distribution of different fluorescently labeled PCPs in mice at various time points: **(A)** Fluorescent PCPs; **(B)** Whole-body distribution; **(C)** Major organs (heart, liver, spleen, lung, kidney; arranged from top to bottom, left to right); **(D)** Stomach; **(E)** Small intestine; **(F)** Cecum and colon.

As shown in Figure 1A; Supplementary Figure S2, fluorescently labeled PCPs exhibited detectable signals under the *in vivo* imaging system, confirming their applicability in tracing the biodistribution of PCPs. After oral administration, fluorescence could be observed within 1–12 h, mainly distributed in the abdominal region, and weakened markedly after 24 h (Figure 1B). Signals in systemic organs remained faint (Figure 1C). The gastric residence time was mainly 1–3 h (Figure 1D), after which the polysaccharides moved into the small and large intestines. Notably, P0 exhibited shorter retention in the small intestine compared with steamed PCPs (Figure 1E). By 6 h, most polysaccharides had reached the cecum and colon, where they persisted for up to 12 h, showing the longest retention among all organs. Thus, the colon, particularly the interaction between polysaccharides and gut microbiota, appears to be critical for mediating the biological effects of PCPs. Taken together, these results indicate that the direct effects of PCPs on systemic organs are limited due to poor bioavailability, whereas their prolonged retention in the colon and interaction with gut microbiota are key factors underlying their biological activity.

### 3.2 Effects of different PCPs on body weight and food intake

During the 8-week intervention, no significant differences were observed among groups in body weight or food intake (Supplementary Figures S3A, S3B), indicating comparable dietary tolerance to PCP supplementation.

### 3.3 Effects of different PCPs on exhaustive weight-loaded swimming time and anti-fatigue related biochemical indexes

**FIGURE 2:**
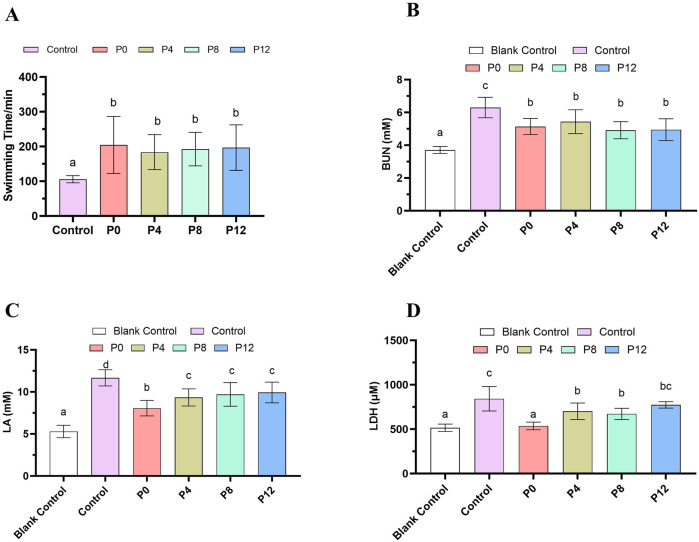
Effects of different PCPs on **(A)** exhaustive weight-loaded swimming time and anti-fatigue related biochemical markers: **(B)** Blood urea nitrogen (BUN), **(C)** lactic acid (LA), **(D)** Lactic dehydrogenase activity (LDH). Different letters represent the statistical differences.

Weight loaded swimming is used to assess anti-fatigue activity of mice (Shen et al., 2021). As shown in Figure 2A, supplementation of PCPs in diet at 0.1% for 8 weeks significantly prolonged the exhaustive swimming time compared with controls, confirming anti-fatigue potential of different PCPs. However, no significant differences s in exhaustive swimming time were detected among the different PCP groups.

To study the potential mechanisms of anti-fatigue effects, biochemical indexes related to fatigue were determined, including blood urea nitrogen (BUN), lactic acid (LA), and lactic dehydrogenase activity (LDH) (Figures 2B–D). When energy supplied by carbohydrates and lipid was insufficient, the proteins were decomposed. The accumulation of BUN is a sign of fatigue, and an increase in BUN further decreases the muscle strength (Zhong et al., 2019). In Figure 2B, swimming exercise significantly increased BUN accumulation compared with rest group. PCPs treatment reduced BUN accumulation. Intensive exercise and anaerobic metabolism can accumulate excessive lactic acids, which causes discomfort involving local muscles, results in fatigue. Compared with rest group, swimming exercise significantly increased lactic acid (LA) content (Figure 2C). All PCP treatments lowered the LA content. LA content in P0 group was significantly lower than that in other PCPs from steamed rhizomes. Higher LDH regulates the metabolism of lactic acid and an indicator of muscle injury, which can permeate into the blood after a long period of exercise of exhaustion. As shown in Figure 2D, LDH was downregulated by PCP treatment, showing similar trends as that of lactic acid. However, the P12 group did not significantly lower the LDH activity, suggesting weak anti-fatigue effects. Thus, all PCPs had anti-fatigue effects by increasing exhaustive swimming endurance. Nevertheless, different steaming durations resulted in changes in their anti-fatigue biochemical indexes.

### 3.4 Effects of different PCPs on fecal SCFAs

**FIGURE 3:**
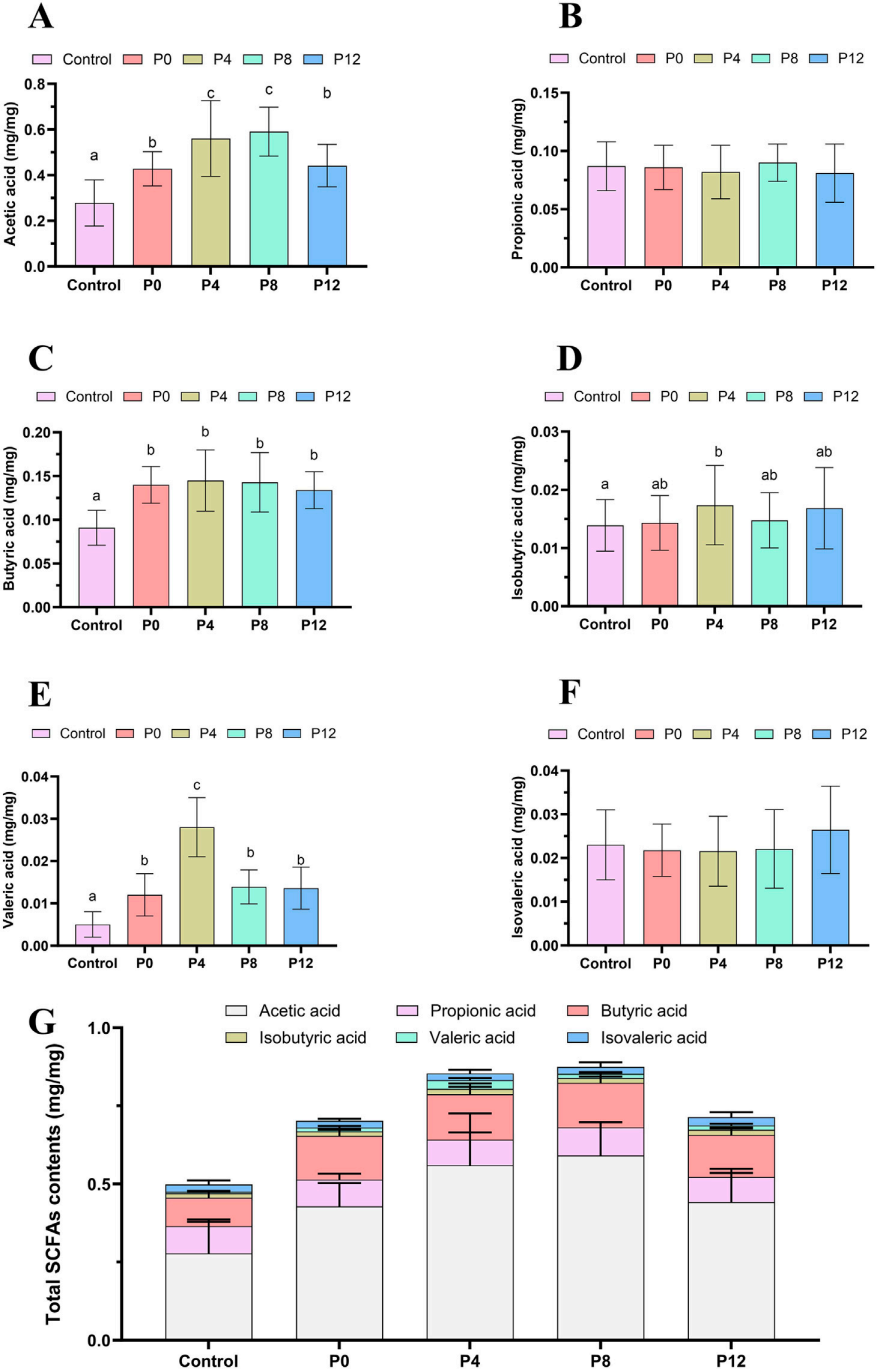
Effects of different PCPs on fecal short-chain fatty acids (SCFAs) in mice: **(A)** acetic acid, **(B)** Propionic acid, **(C)** Butyric acid, **(D)** Isobutyric acid, **(E)** Valeric acid, **(F)** Isovaleric acid, **(G)** Total Short-chain fatty acids. Different letters represent the statistically significant differences, n = 8/group.

As shown in Figure 3, different PCPs promoted the production of intestinal SCFAs, which could participate in the body’s energy metabolism and regulate fatigue (Li Y. et al., 2021; Zhou et al., 2023). The PCP supplementation significantly increased the acetic acid, butyric acid, isobutyric acid, and valeric acid. However, PCPs did not enhance propionic acid and isovaleric acid levels. The P4 group and P8 group showed highest total SCFA production, highlighting the influence of moderate steaming on gut microbial metabolism.

### 3.5 Effects of different PCPs on gut microbiota

**FIGURE 4:**
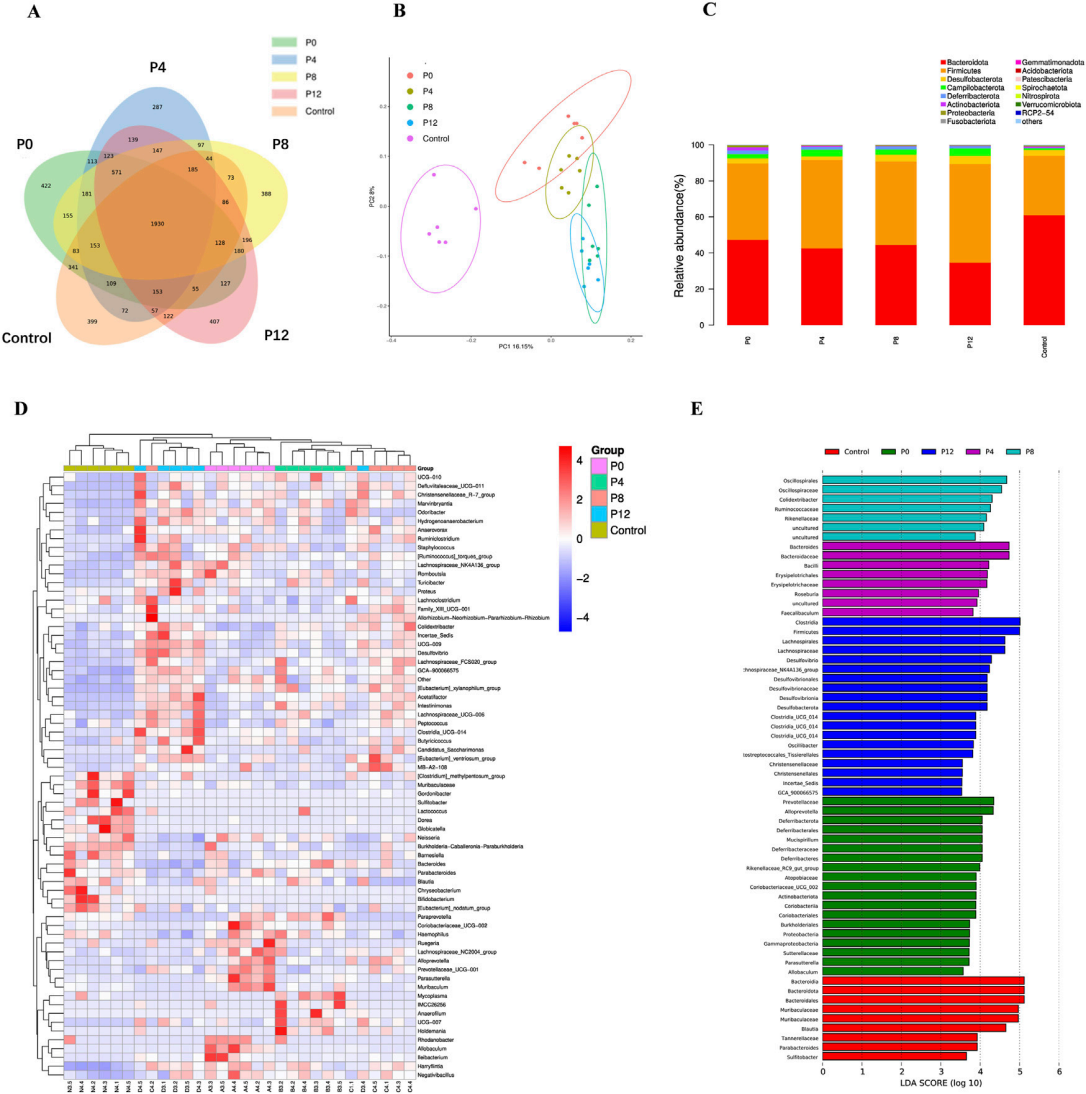
Effects of PCPs on intestinal gut microbiota of mice: **(A)** Venn diagram of OTUs, **(B)** β-diversity (principal coordinate analysis (PCoA) of the unweighted UniFrac distance), **(C)** Gut microbiota in phylum level, **(D)** Gut microbiota in genus level, **(E)** LEfSe of gut microbiota.

The diversity of fecal microbiota composition was analyzed by 16S rRNA gene sequencing. According to Venn diagram (Figure 4A), 1930 common OTUs were observed in five groups. The number of unique OTUs were 422, 287, 388, 407, 399 in P0, P4, P8, P12, and control groups, respectively. Moreover, α-diversity was used to reflect richness, diversity and evenness of species within samples. As shown in Table 1, all PCP supplementation increased α-diversity compared with control group, as indicated by Simpson, Chao1, Shannon, goods-coverage, and PD_whole_tree index. These findings reflect significant compositional differences.

**TABLE 1:** α-diversity in mice gut microbiota.

Groups	Simpson	Chao1	Shannon	Goods_coverage	PD_whole_tree
Control	0.930 ± 0.019^a^	2,521.05 ± 213.12^a^	6.266 ± 0.375^a^	0.990 ± 0.001^a^	64.290 ± 3.720^a^
P0	0.978 ± 0.003^b^	3,047.98 ± 160.50^b^	7.237 ± 0.176^b^	0.988 ± 0.001^b^	75.488 ± 3.375^b^
P4	0.975 ± 0.005^b^	2,820.31 ± 166.88^b^	7.080 ± 0.200^b^	0.988 ± 0.001^b^	71.395 ± 3.698^b^
P8	0.980 ± 0.005^b^	2,875.19 ± 314.51^b^	7.268 ± 0.313^b^	0.988 ± 0.001^b^	71.425 ± 6.467^b^
P12	0.980 ± 0.006^b^	2,918.56 ± 276.80^b^	7.323 ± 0.235^b^	0.988 ± 0.001^b^	72.207 ± 4.224^b^

*Each value represents mean ± SD., The different letters represent the statistical differences at *P* < 0.05. n = 6/group.

The β-diversity, assessed by principal coordinate analysis (PCoA) of the unweighted UniFrac distance (Figure 4B), demonstrated clustering patterns that distinguished PCP groups from controls, with P0 and P4 forming one cluster and P8/P12 forming another. In addition, LEfSe analysis indicated significant differences in microbial groups among the PCP and control groups (Figure 4E). At phylum level, the dominant microbiota were *Bacteroidota* and *Firmicutes*. As shown in Figure 4C; Supplementary Figure S4a, *Bacteroidota* in PCPs groups decreased compared with control group. The P12 group had the lowest proportion of *Bacteroidota*. The proportion of *Firmicutes* in PCPs groups increased with the increase in steaming time. P0 had higher proportion of *Proteobacteria*, while other PCPs from steamed rhizomes did not change the proportion of *Proteobacteria*. Moreover, P0 treatment had higher proportion of *Actinobacteriota* than the other PCPs groups. Additionally, P8 and P12 supplementations significantly decreased the proportion of *Actinbacteria*. At genus level (Figure 4D; Supplementary Figure S4b), the SCFAs-producing bacteria *Roseburia* significantly increased in all the PCPs groups. The P4 group showed highest proportion of *Roseburia*. Moreover, P4 had a higher relative abundance of *Bacterorides* than the other PCP groups. T*he relative abundance of Muribaculaceae was decreased in the all PCPs treatment and P4 showed lowest proportion.* With increase of steaming time, the proportion of Lachnospiraceae*_UCG-006* and *Colidextribacter* increased while the proportion of Rikenellaceae_RC9_gut_group decreased. *Moreover,* P0 had the relatively lower abundance of *Anerotruncus* than the other PCPs groups.

### 3.6 Molecular mechanisms underlying steaming-modulated bioactivity

**FIGURE 5:**
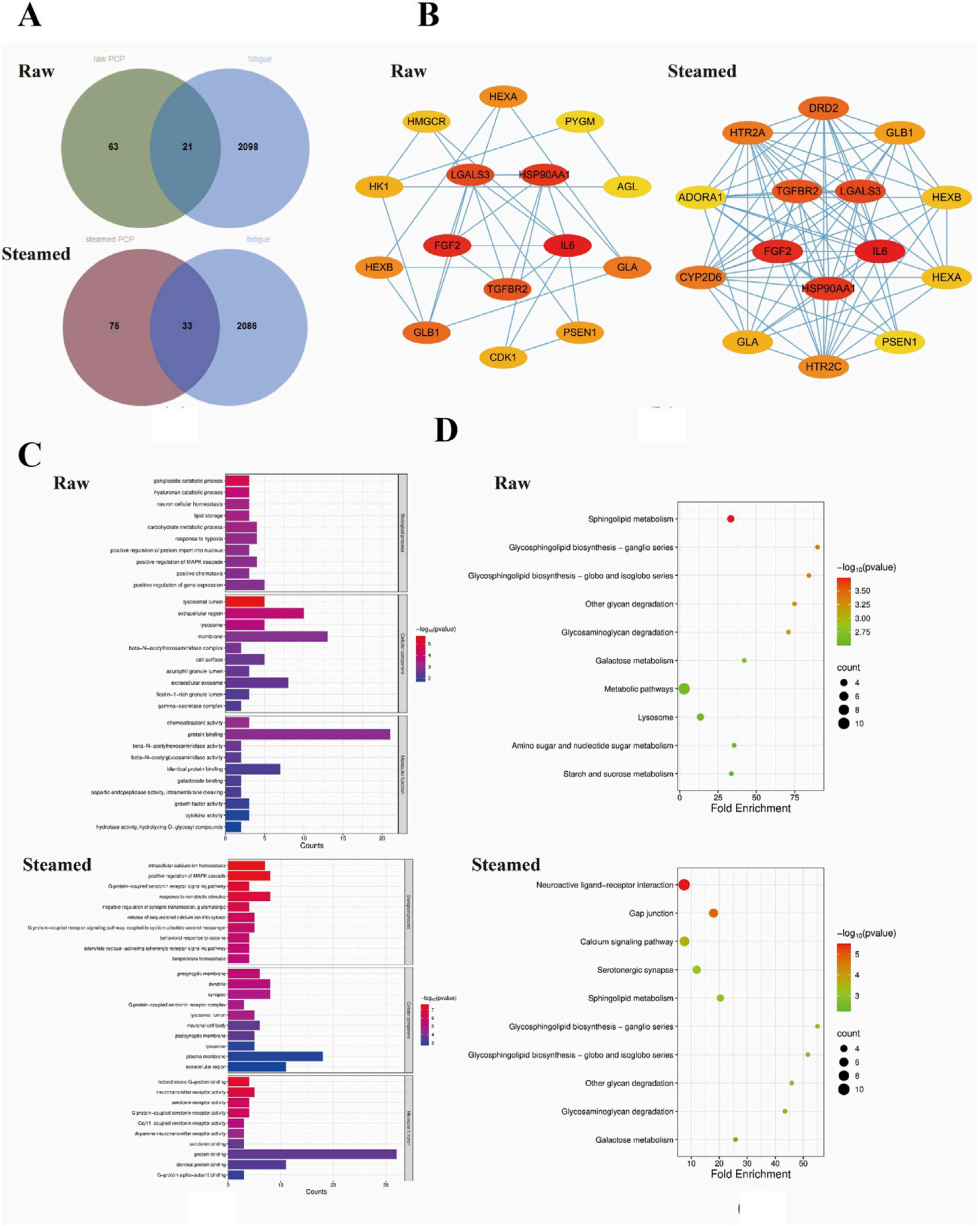
Network pharmacological analysis of PCPs for fatigue. **(A)** Venn plot of the intersection between PCP and fatigue targets, **(B)** PCP and fatigue target intersection PPI network, **(C)** GO analysis, **(D)** KEGG analysis. In all panels, **(A)** represents raw PCP and **(B)** represents steamed PCP.

To decipher how structural changes during steaming remodel PCP bioactivity, network pharmacology analysis was performed. By collecting of multiple databases, 84 drug treatment targets of raw PCP, 108 drug treatment targets of steamed PCP and 2,119 disease targets were obtained. As illustrated by the Venn diagram, 21 common targets in raw PCP and 33 common targets in steamed PCP were obtained by the intersection of the two (Figure 5A). Genes involved in the top enriched pathways were used to construct a PPI network *via* the STRING database, and core genes were further prioritized using the cytoHubba plugin in Cytoscape 3.10.3. Hub genes of raw PCP included targeted metabolic enzymes (*HEXA, HMGCR, PYGM*) and lysosomal regulators (*GLB1, PSEN1*), whereas steamed PCP prioritized neuroreceptors (*DRD2, HTR2A, ADORA1*) and signaling modulators (*TGFBR2, FGF2*) (Figure 5B). In addition, we carried out a functional enrichment study using GO and KEGG on the DAVID database and identified the top 10 pathways (Figures 5C,D). We found that genes of raw PCP were enriched in Sphingolipid metabolism pathway and lysosomal catabolism (e.g., ganglioside catabolism, hydrolase activity, etc.). In contrast, genes of steamed PCP were identified neuroactive ligand-receptor interaction and serotonergic synapses pathways, and GO analysis emphasized G protein-coupled serotonin signaling and dopamine receptor activity. This suggests steaming shifts PCP targeting from peripheral metabolism to neural regulation.

## 4 Discussion

This study demonstrates that PCPs exhibit poor systemic absorption but prolonged colonic retention, with anti-fatigue effects mediated by gut microbiota. Raw PCPs provided stronger anti-fatigue biochemical benefits, while moderate steaming of 4 h enhanced SCFA production. However, prolonged steaming, especially for 8–12 h, weakened the anti-fatigue efficacy and SCFA production. Network pharmacology further revealed distinct mechanisms between raw and steamed PCPs, highlighting the importance of processing in determining their bioactivity.

*In vivo* fluorescence imaging confirmed that PCPs exhibited only weak signals in major organs, consistent with previous reports showing limited translocation of *Polygonatum* polysaccharides (Wu et al., 2024). This indicates that PCPs are unlikely to exert strong direct effects on systemic tissues, as effective concentrations remain low. The human equivalent dose derived from our long-term mouse intervention (100 mg/kg) was 8 mg/kg of polysaccharide. For a 60 kg adult, this corresponds to a daily intake of 4.8–9.6 g of rhizomes (based on a polysaccharide content of 5%–10%), a range consistent with the Chinese Pharmacopoeia (Chinese Pharmacopoeia Commission, 2020). Nevertheless, tissue concentration levels remain limited. P0 displayed shorter colonic retention than steamed samples, likely due to its smaller molecular size, while all PCPs persisted longest in the colon (up to 12 h), emphasizing the role of gut microbiota. Polysaccharides with molecular weights below 12–20 kDa have been shown to cross the intestinal barrier and distribute to organs (Zhang B. et al., 2021; Zhang Y. et al., 2021). While raw PCPs generally have smaller molecular sizes (P0: 334.8 kDa and 7.074 kDa (2 peaks)) and processed PCPs larger ones (P4:1,163 kDa and 77.63 kDa (2 peaks); P8: 112.4 kDa (1 peak); P12: 67.1 kDa (1 peak) (Wu et al., 2022), both raw and steamed PCPs display low absorption efficiency, consistent with previous findings of low bioavailability (4.54%) (Wu et al., 2024; Bi et al., 2022). Overall, the data suggest that primary bioactivity of PCPs is mediated by prolonged colonic retention and microbial interactions rather than systemic uptake.

Although longer steaming did not cause significant difference in the exhaustive swimming time, it has negative impacts on biochemical outcomes. P12 was less effective in lowering LA and LDH levels, whereas raw PCPs showed stronger activity, which may be related to molecular weight. This agrees with earlier reports that smaller molecular fractions (<30 kDa) from polysaccharide of steamed Ginseng of exert anti-fatigue effects, while higher fractions lack activity (Jiao et al., 2021). In addition, structural features (monosaccharide composition, branching, molecular weight) of polysaccharides in association with functional effects (including anti-fatigue), and may account for the differences in biochemical markers observed among the groups (Yang et al., 2025). Furthermore, steaming has been shown to decrease polysaccharide content (Wu et al., 2022), which means that, at the same dosage, the steamed rhizomes contain a lower concentration of polysaccharides. As a result, the amount of active PCPs that the body is exposed to decreases, leading to a reduction in its anti-fatigue efficacy. Thus, from the aspect of anti-fatigue effects, raw rhizomes are preferable, which both has higher polysaccharide content and superior anti-fatigue efficacy. However, direct consumption is limited by throat irritation. Based on these contradictory views, extracting polysaccharides from raw rhizomes, which preserves the active metabolites and enhances their potential as a health supplement. In the other aspect, applying shorter steaming could maximize bioactivity of rhizomes, which is usually consumed, while prolonged steaming (>12 h), common in commercial practice, may reduce effectiveness. Notably, steaming durations exceeding 12 h, such as the “nine steaming, nine drying” process commonly used in commercial products, compromise the bioactivity of *Polygonatum*. Reducing steaming time is therefore crucial, as it helps retain more active ingredients while also lowering energy consumption and improving the cost-effectiveness during processing. On the other hand, steaming has been shown to increase other bioactive metabolites like polyphenols and flavonoids (Guan et al., 2024). Thus, achieving a balance between preserving polysaccharides and enhancing these complementary bioactive metabolites is essential for optimizing the final product’s overall efficacy, and this balance warrants further investigation.

Acetate and butyrate, key SCFAs that contribute to colonic energy metabolism and glucose regulation (Guan et al., 2022), were elevated in all PCP groups, with P4 and P8 showing the highest production. This may partially explain its anti-fatigue effect. Previous studies have similarly demonstrated that mild steaming (e.g., 18 h) enhanced SCFA generation, whereas prolonged steaming (27 h) reduced it in immune-impaired mice (Su et al., 2023). These findings suggest that moderate steaming can promote the probiotic characteristics of PCPs, while excessive steaming exerts a negative impact. Nevertheless, higher SCFA production did not consistently translate into superior anti-fatigue performance, since raw PCPs still exhibited stronger biochemical anti-fatigue benefits. This indicates that SCFAs are contributory but not sufficient determinants of anti-fatigue activity. Molecular weight distribution may further explain these differences: raw rhizomes contain both small- and high-molecular-weight fractions, with the smaller fractions being associated with anti-fatigue effects, whereas steaming beyond 6 h substantially reduced these small fractions, yielding predominantly high-molecular-weight polysaccharides (Wu et al., 2022).

Given that SCFAs alone could not fully explain the differences in anti-fatigue efficacy, alterations in gut microbiota composition provide additional insights. PCP supplementation significantly enhanced microbial α-diversity and induced distinct community shifts compared with controls, which aligns with the reported impact of steaming time on PCP structure (Wu et al., 2022). Previous work also showed that different steaming cycles (6 vs. 9) produced distinct microbiota aggregations and immune responses (Su et al., 2023). In this study, mild steaming (4 h) generated microbial profiles clearly distinct from those of longer steaming (8–12 h), the latter being common in commercial practice. At the phylum level, the Firmicutes/Bacteroidota (F/B) ratio was significantly reduced in P12, a change that correlated with its weakest anti-fatigue performance (Wang Y. et al., 2022; Wei et al., 2023). Furthermore, PCP treatment also decreased Bacteroidetes compared with controls, consistent with clinical observations in chronic fatigue patients who showed lower Firmicutes and Bacteroidetes abundance (Lupo et al., 2021). Steamed PCPs increased *Anaerotrunus*, a genus previously linked to exercise (Zhou et al., 2021), whereas P0 did not, which may help explain the stronger anti-fatigue effects observed in steamed groups. Meanwhile, steamed PCPs reduced Proteobacteria, a shift that could alleviate gastrointestinal irritation associated with raw rhizome consumption, but also decreased Actinobacteria, a group associated with greater anti-fatigue activity (Guan et al., 2022). Moreover, the SCFA-producing bacterium *Roseburia* increased in all PCP groups, with the highest levels observed in the P4 group, which is consistent with the SCFA data (Wang Y. et al., 2022). Lachnospiraceae*_UCG-006* and *Colidextribacter* increased linearly with steaming duration (Feng et al., 2023), while Rikenellaceae also shifted, suggesting that different steaming times reprogram microbial communities in divergent ways. These findings, together with earlier reports that Tibetan turnip polysaccharides modulate *Muribaculaceae* and *Lactobacillus* (Liu L. et al., 2022), indicate that microbial responses vary with processing and may contribute to distinct anti-fatigue mechanisms (Liu C. et al., 2022).

The core targets of steamed PCPs include inflammation-related targets such as IL-6 (Tao et al., 2018; Xiao et al., 2022), DRD2 (Tegowski et al., 2018; Liu Y. et al., 2022), and HTR2A. These targets are involved in the regulation of the IL-6/STAT3-JAK2 signaling pathway. Steaming enhances the acetic acid content among SCFA, which activates GPR41/43 and further suppresses the IL-6/STAT3-JAK2 inflammatory pathway (Maruta and Yamashita, 2020; Ang and Ding, 2016). Inhibition of the IL-6/STAT3-JAK2 pathway helps reduce muscle protein degradation and enhance mitochondrial function, thereby improving exercise endurance and anti-fatigue capacity (Huang et al., 2020). It is proposed that the anti-fatigue effect of steamed PCPs may be partially through the regulation of inflammatory pathways by gut microbiota-derived metabolites, alleviating inflammatory responses and consequently contributing to reduced fatigue.

While alterations in SCFA production and microbial composition partly explained the observed anti-fatigue effects, these factors alone could not fully account for the differences between raw and steamed PCPs. Despite higher SCFA levels, steamed PCPs showed weaker anti-fatigue efficacy than raw PCPs, which may be attributed to critical losses in sphingolipid regulation. The superior activity of raw PCPs may derive from direct activation of HEXA/GLB1-mediated sphingolipid degradation (Wang and Zhou, 2025), which reduces ROS—a key mediator of fatigue—and delays fatigue progression (Nikolova-Karakashian and Reid, 2011). Moreover, lysosomal catabolism preserves calcium release in fatigued muscle by preventing sphingolipid-induced inhibition of SR calcium channels, further supporting anti-fatigue activity (Nikolova-Karakashian and Reid, 2011; Shen et al., 2012). In contrast, steaming redirected PCP targets toward neuroreceptors (e.g., HTR2A) and neuroactive pathways. However, these shifts could not compensate for the loss of PYGM, an enzyme critical for glycogen mobilization and energy supply during muscle contraction (Migocka-Patrzałek and Elias, 2021; Maltese et al., 2016), or for the impaired sphingolipid metabolism. As a result, SCFA-mediated serotonin synthesis alone (Cordeiro et al., 2017; Yano et al., 2015) was insufficient to counteract the deficit in core fatigue-resistance mechanisms, explaining why steamed PCPs were less effective despite enhanced fermentation.

## 5 Conclusion

All PCPs function primarily through gut microbiota interactions rather than systemic absorption, as evidenced by their prolonged colonic retention and limited organ distribution. Both raw and steamed rhizomes showed anti-fatigue effects, but their structural differences led to distinct outcomes. Prolonged steaming (>8 h) leads to a notable reduction in anti-fatigue efficacy, whereas moderate steaming enhanced SCFA production and microbial diversity. The anti-fatigue activity of PCPs can be partly attributed partly to microbiota modulation and SCFA generation. Network analysis further revealed that steaming redirected PCP bioactivity from metabolic regulation to neural signaling, it is speculated that this shift failed to compensate for the loss of PYGM, a key enzyme for glycogen mobilization during muscle contraction, and for impaired sphingolipid metabolism. Collectively, these results underscore the critical importance of controlled steaming protocols in the processing of *P. cyrtonema* to preserve its structural and functional efficacy, providing a scientific basis for optimizing traditional preparation methods.

## Data Availability

The data presented in the study are deposited in the Figshare repository, doi: 10.6084/m9.figshare.30870614.
