# Supplementary material for: Steaming duration-dependent effects on the in vivo distribution, anti-fatigue activity, and gut microbiota modulation of Polygonatum cyrtonema Hua polysaccharides
Source: Front Pharmacol. 2026 Jan 7;16:1721319. doi: 10.3389/fphar.2025.1721319 (PMC12819684; doi:10.3389/fphar.2025.1721319)
Supplement: Supplementary file 1 [file Supplementaryfile1.docx]

Supplementary Materials


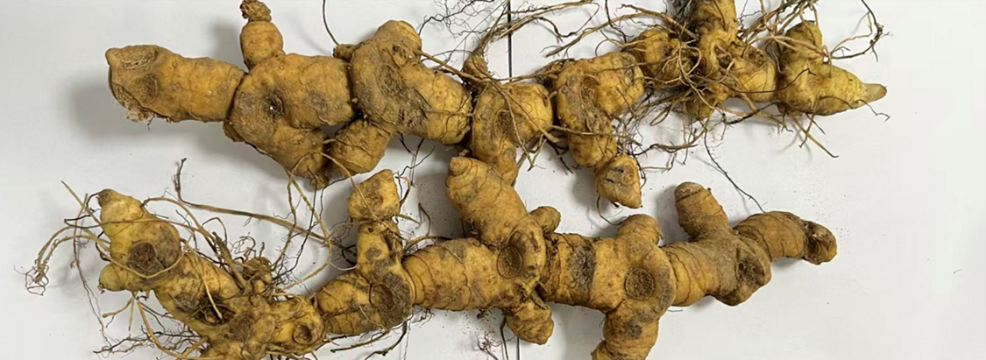


**Supplementary Figure 1.** Raw rhizomes of *Polygonatum cyrtonema* Hua used in this study

**Supplementary Table 1** SCFAs standards

| . | Retention time /min | Linear | R^2^ | Range (mg/mL) |
| --- | --- | --- | --- | --- |
| Acetic acid | 4.67 | Y=662.5X+107.54 | 0.997 | 25-2000 |
| Propionic acid | 5.89 | Y=2980.4X+26.966 | 0.993 | 15.6-500 |
| Butyric acid | 7.54 | Y=3678.9X+12.496 | 0.992 | 15.6-500 |
| Isobutyric acid | 6.38 | Y=4070.2X+19.953 | 0.999 | 3.125-100 |
| Valeric acid | 8.43 | Y=4240.9X+39.693 | 0.994 | 15.6-500 |
| Isovaleric acid | 9.58 | Y=3530.4X+32.402 | 0.999 | 3.125-100 |


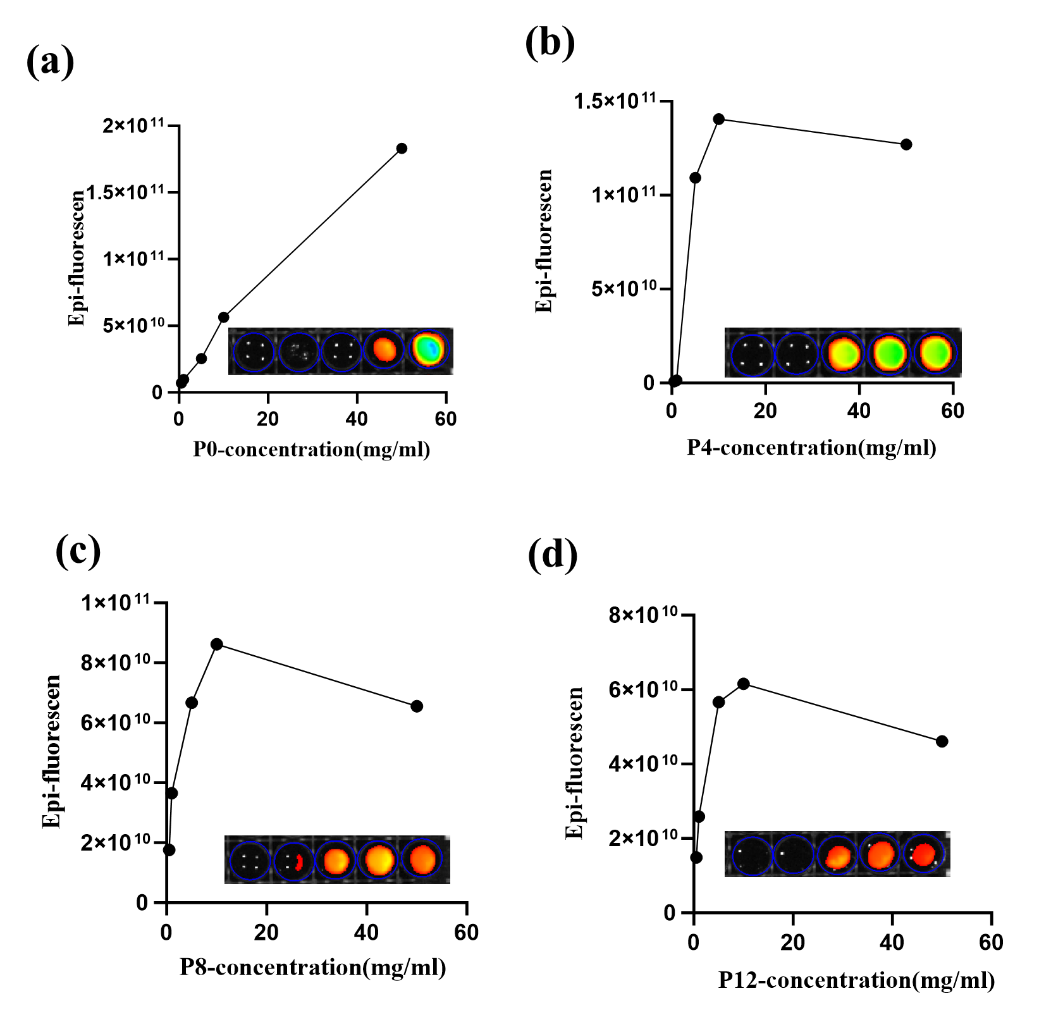


**Supplementary Figure 2.** Fluorescence intensity of FITC-conjugated polysaccharides (FITC-PCPs) derived from different *Polygonatum cyrtonema* Hua Polysaccharides in *in vivo* imaging: (a) P0; (b) P4; (c) P8; (d) P12.

**Supplementary Figure 3.** Effects of different PCPs on (a) body weight; (b) food intake of mice.


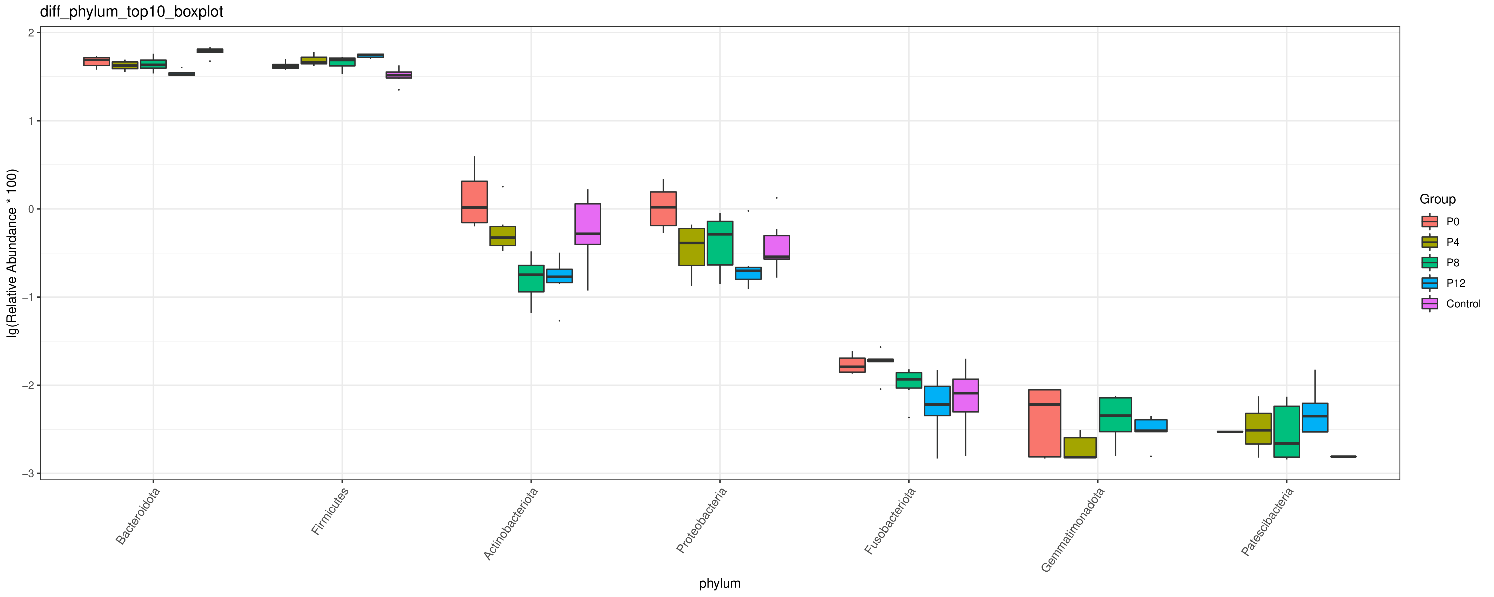


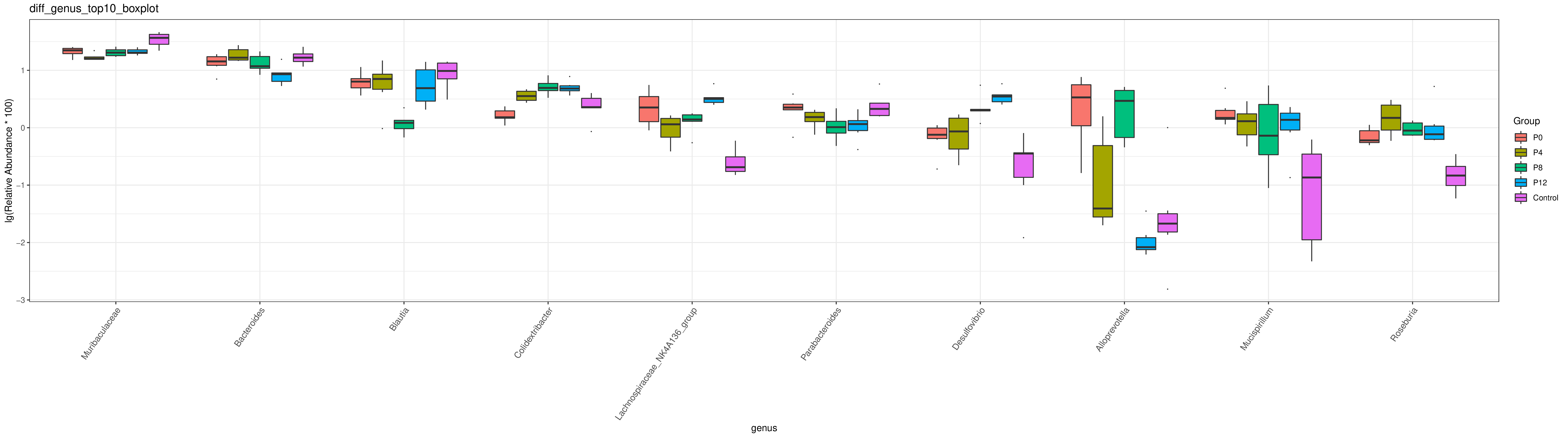


**Supplementary Figure 4.** Effects of different PCPs on gut microbiota structure: (a) top different on phylum; (b) top different on genus.
